# Supplementary material for: Causal Relationship between Aging and Anorexia Nervosa: A White-Matter-Microstructure-Mediated Mendelian Randomization Analysis
Source: Biomedicines. 2024 Aug 16;12(8):1874. doi: 10.3390/biomedicines12081874 (PMC11351342; doi:10.3390/biomedicines12081874)
Supplement: Supplementary file 1 [file biomedicines-12-01874-s001.zip › Supplementary Figure/Figure S4/Singe SNP plot of MR analysis for TL to ubm-b-1718.pdf]

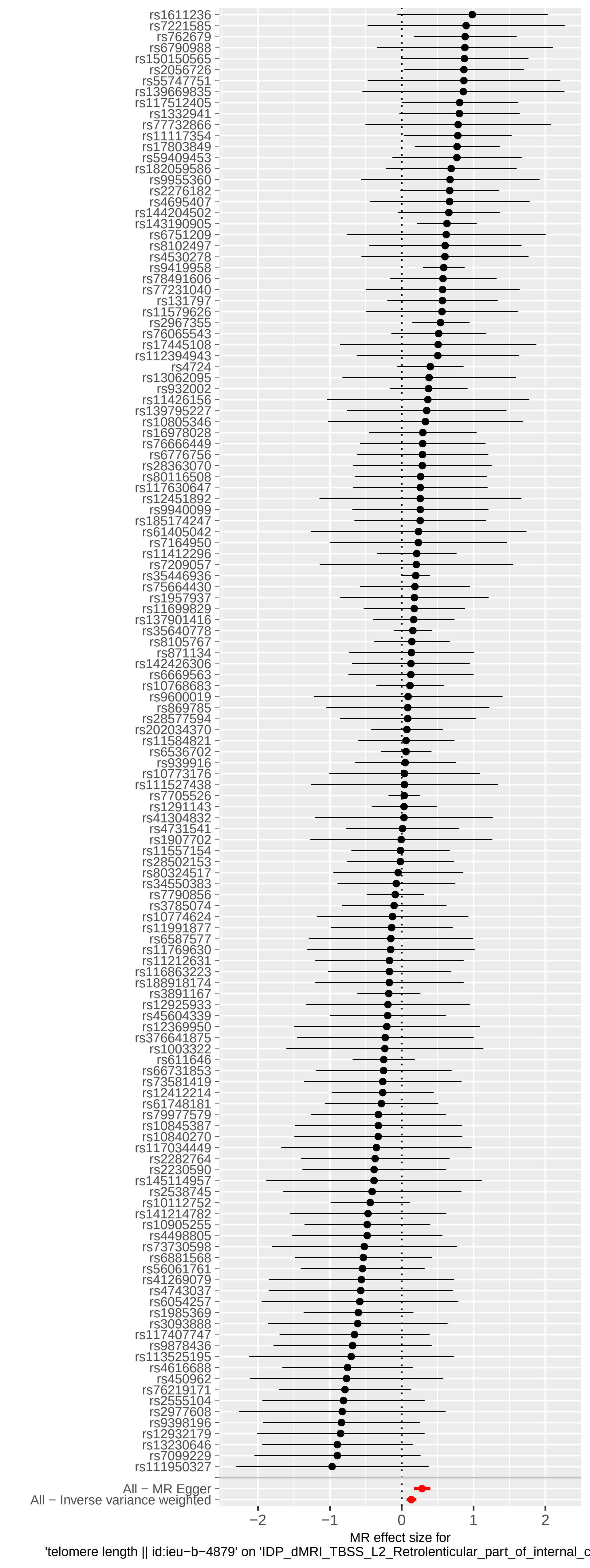

rs1611236
rs7221585
rs762679
rs6790988
rs150150565
rs2056726
rs55747751
rs139669835
rs117512405
rs1332941
rs77732866
rs11117354
rs17803849
rs59409453
rs182059586
rs9955360
rs2276182
rs4695407
rs144204502
rs143190905
rs6751209
rs8102497
rs4530278
rs9419958
rs78491606
rs77231040
rs131797
rs11579626
rs2967355
rs76065543
rs17445108
rs112394943
rs4724
rs13062095
rs932002
rs11426156
rs139795227
rs10805346
rs16978028
rs76666449
rs6776756
rs28363070
rs80116508
rs117630647
rs12451892
rs9940099
rs185174247
rs61405042
rs7164950
rs11412296
rs7209057
rs35446936
rs75664430
rs1957937
rs11699829
rs137901416
rs35640778
rs8105767
rs871134
rs142426306
rs6669563
rs10768683
rs9600019
rs869785
rs28577594
rs202034370
rs11584821
rs6536702
rs939916
rs10773176
rs111527438
rs7705526
rs1291143
rs41304832
rs4731541
rs1907702
rs11557154
rs28502153
rs80324517
rs34550383
rs7790856
rs3785074
rs10774624
rs11991877
rs6587577
rs11769630
rs11212631
rs116863223
rs188918174
rs3891167
rs12925933
rs45604339
rs12369950
rs376641875
rs1003322
rs611646
rs66731853
rs73581419
rs12412214
rs61748181
rs79977579
rs10845387
rs10840270
rs117034449
rs2282764
rs2230590
rs145114957
rs2538745
rs10112752
rs141214782
rs10905255
rs4498805
rs73730598
rs6881568
rs56061761
rs41269079
rs4743037
rs6054257
rs1985369
rs3093888
rs117407747
rs9878436
rs113525195
rs4616688
rs450962
rs76219171
rs2555104
rs2977608
rs9398196
rs12932179
rs13230646
rs7099229
rs111950327
All − MR Egger
All − Inverse variance weighted
−2
−1
0
1
2
MR effect size for
'telomere length || id:ieu−b−4879' on 'IDP_dMRI_TBSS_L2_Retrolenticular_part_of_internal_c
